# Early Perioperative Outcomes of a Newly Designed Metaphyseal Tibial and Femoral Cone in Revision Knee Arthroplasty

**DOI:** 10.7759/cureus.96239

**Published:** 2025-11-06

**Authors:** Brandon K Couch, Allyson N Pfeil, Hari Surnedi, Jeremiah Mekonnen, Anay Patel, Corey F Hryc

**Affiliations:** 1 Research, Fondren Orthopedic Research Institute, Houston, USA; 2 Kinesiology, Rice University, Houston, USA

**Keywords:** metaphyseal cones, osteolysis, patient-reported outcomes (pros), survivorship, total knee revision arthroplasty

## Abstract

Background: Metaphyseal fixation with modern cones or sleeves is often necessitated in revision total knee arthroplasty (rTKA). Survivorship and outcomes data, particularly of novel systems, are limited. This study aims to report the early outcomes and survivorship of a series of rTKA patients receiving a specific tibial or femoral cone.

Materials and methods: A retrospective review of a prospectively collected internal database was conducted for all rTKA patients with a specific metaphyseal cone (ATTUNE, Johnson & Johnson MedTech, Warsaw, Indiana, United States) at a minimum three-month follow-up. Data collected included demographics (age, sex, body mass index (BMI)), postoperative outcomes (length of stay, 30- and 90-day readmissions, complications, reoperations), radiographic subsidence, and Knee Injury and Osteoarthritis Outcome Score for Joint Replacement (KOOS JR) scores preoperatively as well as three months postoperatively. P-values were obtained by t-tests and assessed as significant if p<0.05.

Results: A total of 24 patients were identified with the following revision indications: mechanical loosening (n=9; 37.5%), infection (n=8; 33.3%), instability (n=6; 25%), and arthrofibrosis (n=1; 4.2%). The mean follow-up was 9.3 months. Thirteen tibial cones (54.2%) and 11 (45.8%) femoral cones were implanted. A semi-constrained prosthesis was used in 22 cases, and a hinged prosthesis was used in two cases. The reoperation-free survivorship was 100%. Twelve patients (50%) had two or more postoperative X-rays, and the average subsidence was ≤0.5 mm in both femoral and tibial cone cohorts. One intraoperative complication and two postoperative complications occurred, all unrelated to the tibial cone construct. Statistically significant improvements to the KOOS JR (p<0.001) were observed at three months.

Conclusions: The early short-term outcomes of this novel metaphyseal cone construct are promising, with a 100% survivorship free from reoperation and no complications attributable to the device. Further follow-up is required to determine the long-term durability of these metaphyseal fixation devices.

## Introduction

Despite advancements in technology and the implementation of patient-specific solutions in total knee arthroplasty (TKA) [1,2], a steady proportion of patients remain dissatisfied [3] with the potential need for revision surgery. Common indications for revision TKA (rTKA) include infection, aseptic loosening, instability, bearing wear, periprosthetic fractures, and osteolysis [4]. These diagnoses, unfortunately, increase the morbidity and mortality rates of patients who necessitate rTKA [5-7].

In the revision setting, surgeons are often faced with the challenge of managing diminished bone stock secondary to infection, osteolysis, and/or the removal of prior implants. The severity of bone loss is often described using popular classification systems, such as the Anderson Orthopedic Research Institute (AORI) classification of bone defects, to aid in communication between surgeons and stratification for research purposes [8]. Modular knee systems, sleeves, and cones are options available to address substantial bone loss and enhance the stability of the construct [9]. Metaphyseal cones are of particular interest due to enhanced surgical flexibility, more personalized anatomic solutions, and favorable survivorship in the literature [10,11].

Innovations in metaphyseal cones have led to the evolution of titanium and titanium alloy constructs as successors to earlier tantalum designs. These new constructs demonstrate some biomechanical advantages to tantalum [12], leading to the emergence of new, proprietary systems that incorporate this engineering. This study sought to report early patient-reported and clinical outcomes of a novel, three-dimensional (3D) printed porous titanium alloy tibial and femoral cone constructs used in conjunction with a franchised system. We hypothesized that the early survivorship-related outcomes would be favorable with minimal subsidence, indicating adequate fit and fixation in these more challenging cases.

## Materials and methods

After being granted "Exempt" status by the Institutional Review Board of Texas Orthopedic Hospital (protocol number: TOH266e), a retrospective query was conducted to identify rTKA patients with a specific tibial or femoral metaphyseal cone who also had the accompanying revision knee system (ATTUNE, Johnson & Johnson MedTech, Warsaw, Indiana, United States) from October 2023 to January 2025. All procedures were performed at Texas Orthopedic Hospital in Houston, Texas, which performed 275 rTKAs during the study period. Data were collected on each eligible patient and included demographics (age, gender, race), medical and surgical history (body mass index (BMI), American Society of Anesthesiologists (ASA) score, prior surgeries, Anderson Orthopedic Research Institute (AORI) classification system [8], and preoperative range of motion (ROM)), implant details (constraint, bearing design, stem length), intraoperative (blood loss, complications) and postoperative details (length of stay, 30- and 90-day readmissions, postoperative complications, additional surgery, postoperative ROM), as well as all available radiographs. Additionally, patient-reported outcome measures (PROMs) such as the Knee Injury and Osteoarthritis Outcome Score for Joint Replacement (KOOS JR), 1-100 via a visual analogue scale (VAS), and the Patient-Reported Outcomes Measurement Information System (PROMIS-10) with physical component score (PCS) and mental component score (MCS) were collected preoperatively as well as three, six, and 12 months postoperatively, when available.

Surgical technique

All procedures were performed by one of the five adult reconstruction fellowship-trained orthopedic surgeons at a single, high-volume orthopedic specialty hospital. All procedures followed a standard medial parapatellar approach, with one case requiring a quad snip for appropriate access. Following implant removal, the degree of bone loss was assessed by the surgeon, and the need for metaphyseal fixation was determined. Concentric cones were prepared with a ream-only system, while bi-lobed, tri-lobed, and femoral cones were prepared with a ream and broach system. The remainder of the construct was then prepared using the surgeon's preferred technique. The level of constraint was determined based on the competency of the collateral ligaments and the ability to appropriately balance the knee. Following preparation, trial components were inserted to ensure appropriate position, alignment, fit, and balance. Final implants were then implanted. Cones were press-fit into their appropriate position, and the final femoral and tibial implants were cemented through the cones. Appropriate knee balance, stability, and ROM were evaluated, and the final polyethylene insert was placed to complete the construct. Postoperative rehabilitation protocols are surgeon- and case-specific; patients participated in a formal physical therapy protocol postoperatively focused on early ROM, gait training, and edema control, followed by strengthening and functional activity training.

Surgical details

The majority of cone usage was in the two-component revision setting (n=23; 95.8%); however, one patient was a one-component (tibial) revision (4.2%). Indications varied, including nine for mechanical loosening (37.5%), eight for infection reimplantation at the second stage (33.3%), six for instability (25%), and one for arthrofibrosis (4.2%). The cone size representation was as follows: eight small (33.3%), eight medium (33.3%), four large (16.7%), and four extra-large (16.7%). The cohort consisted of 13 tibial (54.2%) and 11 femoral cones (45.8%). Of the tibial cones, 10 (76.9%) were concentric, two (15.4%) were bi-lobed, and one (7.7%) was tri-lobed. A semi-constrained prosthesis was used in 22 cases (91.7%), while a rotating-hinge prosthesis was used in two cases (8.3%).

Radiographic analysis

Patients with two or more standing postoperative X-rays were measured for cone subsidence and any remarkable radiographic findings by an orthopedic surgery fellow. Tibial cones were measured on the lateral view from the inferior edge of the cone to a fixed anatomical landmark, either a distinct point on the tibial tubercle or the anterolateral corner of the tibial plateau, and consistent between series. Femoral cones were measured from the proximal edge of the cone to the proximal edge of the boss (femoral stem/implant interface). If the cone was not proximal to the boss, the tip of the stem was used in lieu of the boss as the distal reference point.

Data analysis

Patients were analyzed and reported as a single cohort, as well as divided based on the location of the cone (tibia or femur). Descriptive statistics and appropriate statistical tests were conducted. T-tests were performed on continuous data and χ2 on categorical data. All p-values were assessed as statistically significant if p<0.05.

## Results

Patient overview

Patients' average age was 68.4±7.7 years and BMI 32.1±4.7 kg/m^2^, and 16 (66.7%) patients were female (Table 1). White patients were most represented (n=13; 54.2%), followed by African American (n=10; 41.7%) and Hispanic (n=1; 4.2%). Patients had undergone an average of 1.9±1.2 (range: 1-6) knee surgeries before the cone implantation. The ASA score was 2.9±0.3. Preoperative ROM was recorded for 21 (87.5%) patients with a mean flexion of 105.24°±15.20, where two patients had an extension lag of >10°. Of patients with a reported AORI bone loss grade (n=18; 75%), all were at least an AORI type 2, with one (5.56%) type 2A, 11 (61.11%) type 2B, and six (33.33%) type 3.

**Table 1:** Demographics and description of the included patients AORI: Anderson Orthopedic Research Institute; ASA: American Society of Anesthesiologists; BMI: body mass index; CRS: constrained revision system (varus-valgus constrained); LPS: limb preservation system (hinge)

Variable	Tibial (n=13)	Femoral (n=11)
Age in years, mean±SD (range)	71.38±6.92 (61-83)	64.91±7.26 (56-79)
Female sex, n (%)	12 (92.3)	4 (36.4)
BMI (kg/m²), mean±SD (range)	31.4±5.9 (22.5-43.4)	32.8±2.6 (30.5-40.1)
Race		
African American, n (%)	4 (30.8)	6 (54.5)
Hispanic, n (%)	0 (0)	1 (9.1)
White, n (%)	9 (69.2)	4 (36.4)
Prior surgeries, mean±SD (range)	2.08±1.50 (1-6)	1.64±0.67 (1-3)
ASA score, mean±SD	2.85±0.38	2.91±0.30
Indication		
Arthrofibrosis, n (%)	1 (7.7)	0 (0)
Infection, n (%)	4 (30.8)	4 (36.4)
Instability, n (%)	3 (23.1)	3 (27.3)
Mechanical loosening, n (%)	5 (38.5)	4 (36.4)
Constraint		
CRS, n (%)	11 (84.6)	11 (100)
LPS, n (%)	2 (15.4)	0 (0)
Cone symmetry		
Concentric, n (%)	10 (76.9)	-
Bi-lobed, n (%)	2 (15.4)	-
Tri-lobed, n (%)	1 (7.7)	-
Stem length		
30 mm, n (%)	5 (38.46)	0 (0)
50 mm, n (%)	3 (23.08)	6 (55)
80 mm, n (%)	5 (38.46)	5 (45)
AORI		
2A, n (%)	1 (7.69)	0 (0)
2B, n (%)	8 (61.54)	3 (27.27)
3, n (%)	1 (7.69)	5 (45.45)
Not reported, n (%)	3 (23.08)	3 (27.27)

Clinical and radiographic outcomes

The average hospital stay was 1.5±0.5 days (range: 1.0-2.4) (Table 2). At a mean of 62.5±4.95 months postoperatively, the average flexion achieved was 113.54°±19.86, where one patient had an extension of lag >10°. No patients necessitated further surgery on the study knee during the follow-up period, resulting in 100% reoperation-free survivorship at an average of 9.3±5.0 months follow-up (range: 3-18 months). Twelve patients (50%) had two or more postoperative X-rays for subsidence measurements. Of those 12, nine were tibial cones and three femoral cones. Femoral cone subsidence was found to be 0.0±0.7mm (range: -0.5, 0.8), and tibial cone subsidence was found to be 0.5±0.9mm (range: -0.2, 2.3). No radiolucent lines were observed in either subgroup of patients. Two patients did have minor cement extrusion into the soft tissues that was noted by the operating surgeon, as well as the radiographic reviewer, but all components appeared well-fixed without evidence of propagating fracture, so no action was deemed necessary.

**Table 2:** Postoperative outcomes of tibial and femoral cones One patient was readmitted within 30 days and is also included in the 90-day readmission.

Postoperative outcome	Tibial (n=13)	Femoral (n=11)
Length of stay in days, mean±SD (range)	1.38±0.43 (0.96-2.33)	1.57±0.52 (1.08-2.42)
Intraoperative complication, n (%)	0 (0)	1 (9.09)
30-day readmit, n (%)	1 (7.14)	0 (0)
90-day readmit, n (%)	1 (7.14)	0 (0)
Postoperative complication, n (%)	2 (14.29)	0 (0)
Follow-up duration in months, mean±SD (range)	9.15±6.15 (3-18)	8.82±3.68 (3-14)

Complications

One patient (4.2%) experienced an intraoperative fracture of the lateral tibial cortex during cone reaming. The breach was contained with the remaining three cortices intact and thus did not require further attention or additional surgical intervention. The patient did not experience any adverse events or complications following this occurrence.

Another patient (4.2%) was readmitted on postoperative day (POD) 8 (represented in both 30- and 90-day complication rates) for sanguinous wound drainage. The patient underwent a Doppler ultrasound to assess for venous thromboembolism (VTE) or arterial injury, which was negative. The patient was discharged, and the condition resolved without medical intervention.

Two patients (8.3%) experienced a postoperative complication, one being the aforementioned readmit for wound drainage and VTE workup. Another complication was noted seven months postoperatively, when a patient suffered a ground-level fall and sustained a patellar fracture. The fracture was treated nonoperative and went on to heal without persistent pain or functional deficit.

None of the complications, whether intraoperative or postoperative, was suspected to be related to the use of the cone.

PROMs

Preoperatively, 83.3% of patients complied with PROM collection and reported a KOOS JR of 41.3±12.7, VAS 67.9±20.8, PCS 38.2±6.7, and MCS 45.4±7.7 (Table 3). At the study's minimum follow-up of three months, 66.7% of patients recorded PROMs, which resulted in significant improvements to the KOOS JR (58.1±10.8; p<0.001) and VAS (36.4±15.5; p<0.001). To date, 14 and eight patients have concluded the window for six- and 12-month PROM collection, with response rates at 78.6 and 75%, respectively. The KOOS JR and VAS continued to statistically improve at these time points with respect to the preoperative value. No significant improvements have been observed for the PCS or MCS at any time point.

**Table 3:** Longitudinal changes in patient-reported outcomes following tibial, femoral, and combined revisions Statistical significance compared to preoperative using Student's t-test, with p<0.05 as *, p<0.01 as **, and p<0.001 as ***. KOOS JR: Knee Injury and Osteoarthritis Outcome Score for Joint Replacement; MCS: mental component score; PCS: physical component score; VAS: visual analogue scale

Score	Preoperative	3 months postoperative	6 months postoperative	12 months postoperative
Tibia (n)	11	10	4	2
KOOS JR, mean±SD (range)	42.78±12.45 (20.94-59.38)	57.48±8.68^**^ (47.49-73.34)	63.44±17.05^*^ (47.49-79.91)	69.65±14.52^*^ (59.38-79.91)
VAS, mean±SD (range)	62.91±24.92 (30-100)	38.50±12.49^*^ (19-53)	32.00±30.77 (4-66)	22.50±24.75 (5-40)
PCS, mean±SD (range)	38.88±6.54 (26.70-47.70)	42.11±6.26 (34.90-54.10)	43.43±8.92 (32.40-54.10)	52.70±7.07^*^ (47.70-57.70)
MCS, mean±SD (range)	46.18±7.70 (31.30-56.00)	45.56±12.24 (25.10-67.60)	52.85±5.41 (45.80-59.00)	59.00±0.00 (59-59)
Femur (n)	9	6	7	4
KOOS JR, mean±SD (range)	39.55±13.54 (15.94-61.58)	59.12±14.59^*^ (39.63-76.33)	58.89±17.35^*^ (31.31-79.91)	64.93±8.39^**^ (52.47-70.70)
VAS, mean±SD (range)	73.89±13.28 (50-95)	33.00±20.34^***^ (4-61)	41.14±27.64^**^ (0-65)	24.75±8.77^***^ (15-34)
PCS, mean±SD (range)	37.26±7.15 (26.70-47.70)	39.43±8.45 (26.70-47.70)	38.51±6.97 (32.40-47.70)	40.60±8.80 (29.60-47.70)
MCS, mean±SD (range)	44.54±8.00 (31.30-53.30)	47.58±6.45 (36.30-53.30)	44.04±10.56 (25.10-56.00)	49.05±6.19 (41.10-56.00)
Both (n)	20	16	11	6
KOOS JR, mean±SD (range)	41.33±12.70 (15.94-61.58)	58.09±10.80^***^ (39.63-76.33)	60.54±16.52^**^ (31.31-79.91)	66.51±9.50^***^ (52.47-79.91)
VAS, mean±SD (range)	67.85±20.80 (30-100)	36.44±15.46^***^ (4-61)	37.82±27.64^**^ (0-66)	24.00±13.04^***^ (5-40)
PCS, mean±SD (range)	38.15±6.69 (26.70-47.70)	41.11±7.00 (26.70-54.10)	40.30±7.69 (32.40-54.10)	44.63±9.77 (29.60-57.70)
MCS, mean±SD (range)	45.45±7.67 (31.30-56.00)	46.32±10.23 (25.10-67.60)	47.25±9.77 (25.10-59.00)	52.37±7.03 (41.10-59.00)

Tibial vs. femoral cones

Comparing tibial and femoral cone patients, tibial cone patients were statistically older (71.4±6.9 vs. 64.9±7.3; p<0.05) with more females (92.3% vs. 36.4%; p<0.01) than femoral cone patients (Table 1). There were no differences (p>0.05) regarding the number of prior surgeries, length of stay, follow-up duration, or any preoperative PROMs.

## Discussion

This study sought to report short-term outcomes in a cohort of patients who underwent rTKA using a novel, 3D printed porous tibial or femoral cone. Short-term outcomes were found to be excellent, with patients reporting significant improvements in their PROMs and no patients requiring reoperation during the study period.

Complications

One patient experienced an intraoperative fracture of the lateral tibial cortex during cone reaming. The breach was contained with the remaining three cortices intact and thus did not require further attention or additional surgical intervention. The patient did not experience any adverse events or complications following this occurrence.

Another patient was readmitted on POD 8 (represented in both 30- and 90-day complication rates) for sanguinous wound drainage. The patient underwent a Doppler ultrasound to assess for VTE or arterial injury, which was negative. The patient was discharged, and the condition resolved without medical intervention.

Two patients experienced a postoperative complication, one being the aforementioned readmit for wound drainage and VTE workup. Another complication was noted seven months postoperatively, when a patient suffered a ground-level fall and sustained a patellar fracture. The fracture was treated nonoperatively and went on to heal without persistent pain or functional deficit.

Present findings and literature comparison

To our knowledge, this is the first study to report on this particular metaphyseal cone; however, the utilization of cones for substantial bone loss in rTKA has been in practice since 2002 [13,14]. Our results of significant early improvements in KOOS JR and VAS scores are consistent with prior findings, demonstrating the responsiveness of joint-specific PROMs in the revision setting [15,16]. By contrast, no significant change in PROMIS-10 PCS or MCS was observed, which may reflect the limited sensitivity of global health instruments in small cohorts or the short follow-up window [17]. The minimal subsidence observed (<0.5 mm) is consistent with prior reports of metaphyseal cones in revision TKA, which show early radiographic fixation without apparent subsidence or loosening [18].

Long-term data is now available on initial tantalum designs, with a study reporting a 10-year 97% survivorship free from aseptic loosening requiring the removal of the cone construct [14]. The same study did find a remarkably lower survivorship free from any-cause reoperation of 58%. Although unsurprising, this difference emphasizes the complexity of treating patients with extensive bone loss in the revision setting. Survivorship free from cone construct removal, any revision, and any reoperation were all significantly lowered if the index revision was indicated for infection [14]. This finding makes describing the true impact and any perceived benefit of metaphyseal cones difficult, with so many factors able to contribute to failure.

Another study, also reporting on a similarly newly designed 3D printed porous cone, recognized that this complexity yielded "modest" reoperation-free survival at 86% at a minimum of two years [11]. To address this, they reported on the aseptic loosening-free survivorship as the primary outcome most relevant to the cone performance, which was 100%. Cone-specific fracturing complications (1.3%) were also reported and compared to the work of Tetreault et al., with a cone-specific fracture rate of 2% [11,19]. In our series, complication rates in this series were low and not attributable to the cone construct, which is comparable to the 1-3% cone-specific fracture rate reported in larger series [19].

Limitations

This study is not without limitations. As a case series of 24 patients at a single site, the generalizability of the results may be limited. This study provides a minimum of three-month findings and outcomes on a small group of patients receiving a newly designed cone, which renders the findings preliminary. Further research is certainly necessary to determine the long-term outcomes of this novel system in a larger sample of patients. Additionally, the study was performed in a retrospective fashion and vulnerable to the limitations inherent to this study design. Specifically, the AORI classification was determined through the interpretation of findings in the operative reports, which is dependent on the accuracy and reliability of the surgeon's descriptions. Inconsistency and/or omission of the degree of bone loss in the reports makes accurate reporting difficult. Further research is underway with surgeons classifying bony defects immediately postoperatively to mitigate errors in interpretation and/or omission.

## Conclusions

Device evolution in rTKA continues to change practice and warrant evidence-based research. This study demonstrates that use of the novel 3D printed metaphyseal cone in rTKA was associated with favorable early radiographic and functional outcomes, with no device-related complications at an average of nine-month follow-up. While these findings support short-term safety and stable fixation, longer-term data and larger comparative studies are necessary to determine true efficacy and durability.
